# Effects of spatial consistency and individual difference on touch-induced visual suppression effect

**DOI:** 10.1038/s41598-018-35302-w

**Published:** 2018-11-19

**Authors:** Souta Hidaka, Yosuke Suzuishi, Masakazu Ide, Makoto Wada

**Affiliations:** 1 Department of Psychology, Rikkyo University, 1-2-26, Kitano, Niiza-shi, Saitama 352-8558 Japan; 2 Developmental Disorders Section, Department of Rehabilitation for Brain Functions, Research Institute of National Rehabilitation Center for Persons with Disabilities, 4-1, Namiki, Tokorozawa-shi, Saitama 359-8555 Japan

## Abstract

Crossmodal studies have reported not only facilitatory but also inhibitory perceptual interactions. For instance, tactile stimulation to the index finger of a hand leads to the degradation of visual discrimination performance (touch-induced visual suppression, TIVS). It has been suggested that the magnitude of TIVS depends on the spatial congruency of visuo-tactile stimuli and on individual differences in task performance. We performed a detailed investigation of the effects of spatial consistency and individual differences on the occurrence of TIVS. The visual target and tactile stimulus were presented at co-localized, ipsilateral but not co-localized, or contralateral positions. The degree of autistic traits has been reported to be well variable among the general population and to reflect differences in sensory processing. Therefore, we assessed the magnitude of autistic traits using the autism-spectrum quotient (AQ) as an index of individual differences. TIVS occurred particularly at the ipsilateral but not co-localized position. In contrast, the magnitude of the TIVS was positively correlated with the AQ score when the stimuli were presented at the co-localized position. These findings suggest that the occurrence of TIVS can be modulated both by the spatial relationship between the visual and tactile stimuli and by individual differences in autistic traits.

## Introduction

Our perceptual systems integrate crossmodal inputs in order to establish coherent and robust perceptions of our surrounding environment^1^. In accordance with this view, studies on crossmodal interactions have predominantly reported facilitatory effects of crossmodal inputs on perceptual processing^2,3^. However, it has been reported that both facilitatory and inhibitory processes play important roles in our perception^4^. In fact, some studies have demonstrated that suppressive interactions can also occur between crossmodal inputs. Neurophysiological studies have found that the pooling of neural signals from audiovisual stimuli induces not only facilitatory but also suppressive neural activities in the superior colliculus of cats^5,6^. Human brain imaging studies have also reported that auditory stimuli can induce inhibitory responses in the visual cortex, and vice versa^7^. Similarly, tactile stimulation to the hand was found to inhibit neural responses in visual cortical areas^8,9^.

A human behavioral study reported that performances in tactile distance discrimination on the forearm improved when individuals saw their forearm immediately before rendering a judgment^10^. In a similar experiment, it was further shown that visual information regarding a participant’s body part induced suppressive effects when the participant performed a tactile detection or discrimination task for stimuli presented above the detection threshold^11^. Top-down modulation^10^, including attention^12–14^, may be involved in these visuo-tactile interactions.

Our recent study demonstrated a tactile suppressive effect on visual perception^15^. We presented a tactile vibration to the index finger of each participant’s left hand. The participants were then asked to judge the orientation of a visual target stimulus. We found that the tactile stimulation degraded the visual orientation discrimination performance for visual stimuli (touch-induced visual suppression; TIVS). A key factor for the occurrence of TIVS is the difference in the perceptual intensity of the stimuli^15^: TIVS occurred predominantly when visual targets were presented very briefly at a very weak to just perceptible threshold contrast level, against a clearly perceptible tactile stimulus. This finding is in line with reliability-based interactions of crossmodal perception^16,17^. It has been shown that, in interactions between a visually presented hand image and vibrotactile stimulation, the reliability of each input modulated not only behavioral performance, but also neural responses in the secondary somatosensory area and the connectivity weight between the multisensory cortical area (intraparietal sulcus) and the secondary somatosensory cortical area^18^. Furthermore, TIVS clearly occurred when the tactile and visual stimuli were temporally congruent. The TIVS also occurred when the tactile and visual stimuli were presented ipsilaterally. A functional magnetic resonance imaging (fMRI) study investigating the neural mechanisms underlying TIVS for the tactile and visual stimuli presented at an ipsilateral left position showed larger inhibitory responses in the anterior region of the right visual cortex (V1 and V2) in participants with greater TIVS magnitudes^19^. Activation in the right anterior superior temporal region, including the secondary somatosensory cortical area, was more strongly related to the inhibitory responses in the visual cortex (V1 and V2) for the participants with greater TIVS magnitudes. These findings suggest that TIVS occurs at the perceptual processing stage, and that inhibitory neural modulations, from the somatosensory to the visual cortices, are involved in the occurrence of TIVS.

The spatial congruency aspect of TIVS was demonstrated such that the discrimination performance of the visual target presented on the left side of a display relative to a fixation point was degraded when the tactile stimulus was presented to the index finger of the participant’s left hand^15^. In this situation, the stimuli were presented at an ipsilateral position, but spatially separated (>25 cm). The visual target was presented on a display in front of the participant while her or his hand was located on a desk close to their body. Crossmodal studies have consistently reported that the magnitudes of crossmodal interactions become more evident when stimuli are presented in a spatially co-localized manner^2,3^. This suggests that TIVS could also occur when the visual and tactile stimuli are presented at spatially co-localized positions. In contrast, crossmodal perceptual suppression from the auditory to the visual modality has been reported to show the opposite result^20^. The suppression of visual percepts induced by sounds, which has phenomenal characteristics very similar to those of TIVS, predominantly occurred when the visual and auditory stimuli were presented at an ipsilaterally but spatially distant position: The visual stimuli were presented on the left side of a visual display and the auditory stimuli were presented via the left speaker on headphones placed on the participant’s head. The suppression did not occur when the auditory stimuli were presented from a speaker located at the same position as the visual display so that the visual and auditory stimuli were presented in a spatially co-localized manner. The findings regarding the sound-induced visual suppression suggest that it would be optimal for the occurrence of crossmodal perceptual suppression where crossmodal stimuli are presented as spatially related but not firmly consistent in space, inconsistent with the simple spatial co-localization rule^20,21^. It is therefore necessary to further investigate whether the TIVS occurs when the visual target and the tactile stimulus are presented at a co-localized position.

It is perhaps also noteworthy that an fMRI study of TIVS reported individual differences in the magnitude of TIVS^19^. In this study, participants were initially asked to perform a visual orientation discrimination task without the presentation of a tactile stimulus (baseline condition). The parameters of the visual target were adjusted in order to achieve an orientation discrimination performance of around 70% of correct responses level for each participant. In the subsequent experimental session, participants completed the orientation discrimination task for visual targets whose parameters were set to around the 70% discrimination level, with or without tactile stimulation. TIVS occurred in participants who were able to maintain a discrimination performance level above 50% for the baseline condition in the main experiment. On the other hand, TIVS was not observed in participants whose discrimination performance in the baseline condition was below 50%. To better understand the underlying mechanisms of TIVS, it is necessary to determine whether and how individual differences affect the occurrence of TIVS, by excluding artificial instabilities in behavioral performance and by adopting a reliable index of individual differences.

**Figure 1:**
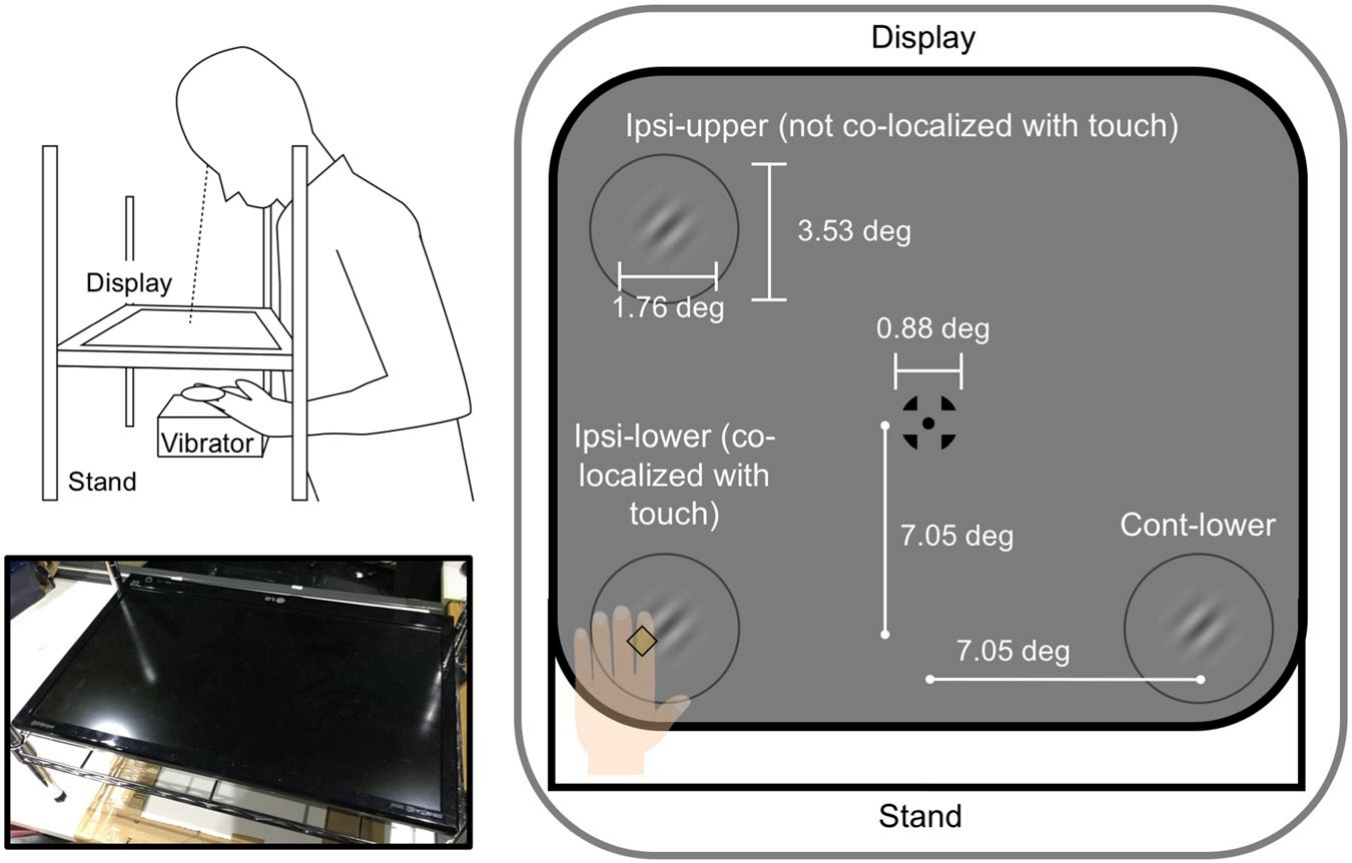
Schematic illustrations of the experimental setup. The tactile stimulus was presented to the index finger of the participant’s left hand, which was placed beneath the display. The visual stimuli were presented on the display, which was placed horizontally in front of the participant, at either co-localized, ipsilateral but not co-localized, or contralateral positions relative to the participant’s left hand.

The purpose of the current study was to investigate the effects of spatial consistency and individual differences on the occurrence of TIVS. To address spatial consistency, we deliberately manipulated the spatial position of the visual target relative to that of the tactile stimulus. A visual display was placed horizontally in front of the participant. We asked the participant to place her or his left hand beneath the display. We presented the tactile stimulus to the index finger of each participant’s left hand (Fig. 1). The visual target stimulus was presented at a position either directly above the participant’s left hand (co-localized), ipsilateral to the hand but separated in depth, or contralateral to the hand.

To investigate individual differences, we focused on autistic traits. Autism spectrum disorder (ASD) is a type of neurodevelopmental disorder, with typical symptoms including deficits in social actions (communication and interaction) and in behavior (restricted and repetitive interests)^22^. Sensory irregularities are also reported in ASD^23^, and have received recent attention from a diagnostic perspective^22^. Irregularities in crossmodal interactions/integration have been reported in individuals diagnosed with ASD^24,25^. For example, individuals diagnosed with ASD have been reported to have a weaker temporal integration of visual and interoceptive (heartbeats) signals when compared to those without diagnosis. This reflects the irregularity of temporal processing between exteroceptive and interoceptive inputs in individuals with ASD^26^. ASD-like characteristics are now considered common properties among the general population rather than ones unique to those with the diagnosis^27,28^. Such characteristics are thought to exist on a continuum among the general population^29–31^. With this view in mind, the Autism-Spectrum Quotient (AQ) has been developed as a measure to assess autistic traits among typical-intelligence individuals both with and without ASD diagnoses^29,32,33^. The score of 33 is considered the cut-off for discrimination of ASD^29^. The particular characteristics of neural and perceptual processing have been studied in general populations along with AQ scores in the contexts of functional connectivity in the brain^34^ and sensory experiences^35,36^. Recent behavioral studies have also reported differences in perceptual performances along with AQ scores both in single (vision)^37,38^ and crossmodal^39–43^ modalities in people without ASD diagnoses. The fMRI study described above, which was carried out to investigate the neural mechanism underlying TIVS, suggests that inhibitory neural modulation from tactile to visual areas increased in parallel with greater TIVS magnitude^19^. The autistic brain is characterized by imbalances in excitatory and inhibitory neural systems^44,45^. Considerable variability in long-range connections in the brain have also been reported in individuals with ASD^46^. Based on these evidences, we considered that autistic traits estimated by AQ could be a reliable measurement to investigate individual differences in the visuo-tactile interactions underlying TIVS.

The current study investigated the effects of spatial consistency on the occurrence of TIVS, using the measurement of autistic traits as an index of individual differences. We adopted the method of constant stimuli to estimate the threshold level and the tactile effect simultaneously^15^, rather than using a predetermined threshold level in each participant^19^. This enabled us to exclude the potential involvement of artificial instability in participants’ performance, namely changes in perceptual performance after the predetermination of the threshold level. Tasks such as a simple detection or speeded response tasks were considered to involve top-down effects such as response/decisional bias (e.g., the presentation of the tactile stimulus induces frequent or speeded responses/judgments). Therefore, we adopted a visual orientation discrimination task in which the tactile stimulus would not be considered to play a role in response/decisional cues in order to isolate the perceptual effect, as in the previous study^15^.

Visuo-tactile interactions are considered to occur based on neural mechanisms having common receptive fields for both visual and tactile stimuli^47,48^. Crossmodal interactions has also been well observed when multisensory stimuli are presented in a spatially co-localized manner^2,3^ and near one’s body^49,50^. Based on these findings, we hypothesized that TIVS could occur when visual and tactile stimuli are presented in a spatially co-localized position. On the contrary, we also expected that the co-localized presentation of the tactile and visual stimuli would not be optimal for TIVS based on the null effect of sound-induced visual suppression^20^ and the task-dependent characteristics for the spatial co-localization rule for crossmodal interactions^21^. It has been reported that individuals diagnosed with ASD display weaker visuo-tactile interaction^43,51–53^. Thus, we predicted that the magnitude of TIVS would decline with increases in the autistic traits.

## Results

We presented the tactile stimulus to our participants (N = 28) as a vibration (300 Hz sinusoidal burst) lasting for 200 ms. The vibration device was attached to the participant’s index finger on the palm of the left hand. A fixation point was presented at the center of the display as a visual stimulus (Fig. 1). A gray ring was presented to the lower left (co-localized with the tactile stimulus position; ipsi-lower condition), the upper left (ipsilateral but not co-localized with the tactile stimulus position; ipsi-upper condition), or the lower right (contralateral to the tactile stimulus position; cont-lower condition) of the fixation point at a distance of 9.97°. A Gabor patch was presented as a target within the ring. Stripes on the target stimulus were slanted either to the left (−45°) or right (+45°). The target’s contrast (Weber contrast) varied from 0.05 to 0.30 in 0.05 steps. We asked the participants to place the index finger of her or his left hand just beneath the visual ring at the lower left position. Following the presentation of the fixation point and the gray ring, the ring changed color from gray to black as the cue for target onset, after which the visual target was presented for 50 ms. Concurrently, the tactile stimulus was presented for 200 ms (touched condition). The onset timings of the visual and tactile stimuli were consistent. Trials without the tactile stimulus were also included to obtain baseline measurements (without-touched condition). After the target presentation, the participants were asked to report the perceived orientation of the target (tilted to the left or right). Each spatial condition was introduced in a blocked design.

After the experiment, we measured the participants’ autistic traits using the Japanese version of the AQ^32^. The AQ is a self-reported questionnaire that contains 50 items describing autistic traits. Participants are asked to rate the degree to which the content of each item describes them, on a 4-point Likert scale (“definitely agree,” “slightly agree,” “slightly disagree,” and “definitely disagree”). Items that the participants rated as 1 or 2 (or 3 or 4, for reversed items) were given a score of 1 point. The scores of all 50 items were summed to calculate the total AQ score.

**Figure 2:**
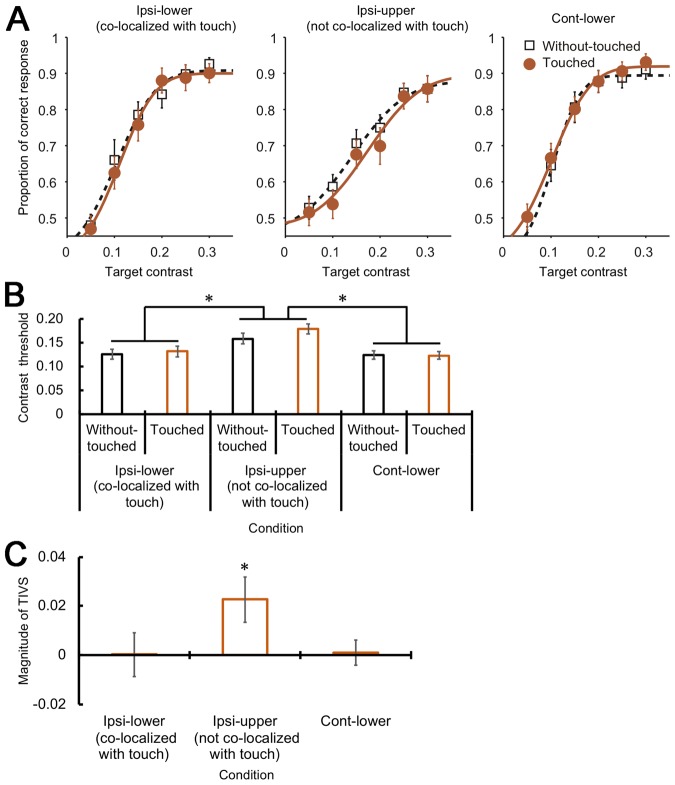
Magnitude of TIVS. (**A**) Psychometric functions obtained for each spatial and tactile condition. The horizontal axis denotes the contrast of the visual targets and the vertical axis denotes the proportion of the correct responses in the visual orientation discrimination task. (**B**) Estimated contrast thresholds. The horizontal axis denotes the conditions and the vertical axis denotes the contrast thresholds. (**C**) The magnitudes of TIVS calculated by subtracting the contrast threshold in the without-touched condition from that in the touched condition. The horizontal axis denotes the spatial conditions and the vertical axis denotes the magnitude of TIVS. Error bars denote the standard errors of the mean (N = 28). Asterisks denote significant differences (*p* < 0.05).

We plotted the proportions of correct responses according to each target’s contrast, for each condition. Cumulative Gaussian functions were fitted to each dataset. With regard to the psychometric functions for each spatial and tactile condition (Fig. 2A), we estimated the 75% response point as the discrimination threshold (Fig. 2B). The threshold of the ipsi-upper condition appeared to be higher (lower visual discrimination performance) than those for the other conditions, irrespective of the tactile condition. A two-way repeated analysis of variance (ANOVA) with the spatial and tactile conditions as factors revealed a significant main effect of the spatial condition (*F*(2, 54) = 18.36, *p* < 0.001, ${n}_{p}^{2}=0.41$). A post hoc test (*p* < 0.05) showed that the threshold for the ipsi-upper condition was significantly higher than those for the other conditions. The main effect of the tactile condition (*F*(1, 27) = 3.66, *p* = 0.07, ${n}_{p}^{2}=0.12$) and the interaction (*F*(2, 54) = 1.57, *p* = 0.22, ${n}_{p}^{2}=0.06$) were not significant.

The main purpose of the analyses was to investigate the magnitude of TIVS for each spatial condition. Thus, we calculated the difference in the thresholds between the tactile conditions (touched – without-touched) as an index of the magnitude of TIVS for each spatial position (Fig. 2C). Larger values indicate stronger TIVS effects. The TIVS magnitude appeared to be greater than zero only for the ipsi-upper condition. A planned one-tailed *t*-test (*p* < 0.05) between the TIVS magnitude and zero found a significant difference for the ipsi-upper condition (*t*(27) = 2.06, *p* = 0.02, *d*_*Z*_ = 0.39). No significant differences were observed for the ipsi-lower or cont-lower conditions (*t*(27) = 0.68 and −0.23, *p* = 0.25 and 0.59, *d*_*Z*_ = 0.13 and −0.04, respectively).

**Figure 3:**
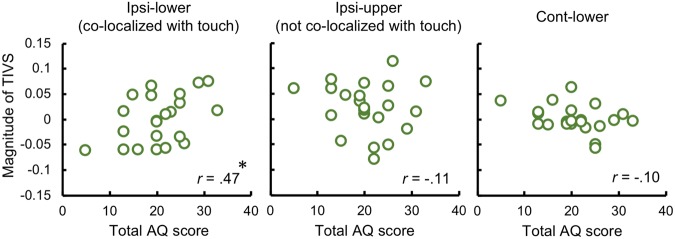
Relationships between the magnitude of TIVS and autistic traits. Scatter plots depict the magnitudes of TIVS and the AQ total scores for each spatial condition. An asterisk denote a significant difference (*p* < 0.05).

With respect to the total AQ score, we performed correlation analyses (two-tailed, *p* < 0.05) of the magnitudes of TIVS and the total AQ score for each spatial condition (Fig. 3). A significant positive correlation was observed between the TIVS magnitude for the ipsi-lower condition and the total AQ score (*r* = 0.47, *p = *0.01), indicating that the magnitude of TIVS increases along with that of the autistic traits. The correlations were not significant for the ipsi-upper or cont-lower conditions (*r* = −0.11 and −0.10, *p* = 0.57 and 0.63, respectively).

## Discussion

In the current study, we investigated the effects of spatial consistency and individual differences on the occurrence of TIVS. The orientation discrimination performance for the visual target was measured at the co-localized (ipsi-lower condition), ipsilateral but spatially separate (ipsi-upper condition), or contralateral (cont-lower condition) positions relative to the tactile stimulus on the index finger of each participant’s left hand. We also measured autistic traits using AQ as an index of individual differences.

Dominant crossmodal interactions have been demonstrated when crossmodal stimuli are presented in a spatially co-localized manner^2,3^ and near one’s body^49,50^. Visuo-tactile interactions have also been reported to occur around common receptive fields for visual and tactile stimuli^47,48^. Thus, one of our predictions was that TIVS would occur preferentially when visual and tactile stimuli were presented at a co-localized position. However, we did not detect TIVS when the stimuli were co-localized (ipsi-lower condition). Rather, TIVS was clearly observed at the ipsilateral but spatially distinct position (ipsi-upper condition) (Fig. 2C).

Consistent with the idea that crossmodal interactions occur based on the reliability of inputs^16–18^, one of the key factors for the occurrence of TIVS is the difference in the perceptual intensity of the stimulus: Visual stimuli with weaker magnitude are suppressed by the clearly perceptible tactile stimulation^15^. In this study, visual discrimination performance was lower (higher contrast threshold) for the ipsilateral but spatially distinct position than for the other spatial conditions irrespective of the tactile condition (Fig. 2B). This may simply be due to better visual performances in the lower visual field^54^. Additionally, the co-localized presentation of the tactile and visual stimuli could not be optimal for the occurrence of TIVS. It has been suggested that characteristics of the spatial co-localization rule for crossmodal interactions are task dependent^21^. The majority of crossmodal interactions in the co-localized situation have been reported as facilitatory effects^2,3^. On the other hand, TIVS demonstrated a perceptual suppressive effect. TIVS and sound-induced visual suppression have been reported when the visuo-tactile stimuli were presented at an ipsilateral but spatially apart position^15,20^. Based on these ideas, we can assume that the ipsilateral but spatially distinct position is an optimal situation for the occurence of TIVS in the current study.

One might assume that the tactile stimulation near the body captured the participant’s attention, leading to degradation of visual performance at the ipsilateral upper position where the visual performance level was relatively lower. In fact, crossmodal attentional capture effects in space have been demonstrated^55^. Crossmodal attentional effects are also reported to be dominant when the attentional cueing stimuli preceded the other stimuli^56,57^. In the current study, however, we presented the visual and tactile stimuli concurrently. Our previous studies also consistently indicate that crssmodal perceptual suppressions occurs specifically when the stimuli are presented without any temporal gaps^15,20^. The inconsistency in the temporal characteristics suggests that the crossmodal attentional capture effect cannot fully explain the results of the present study. Utilization of the indirect perceptual task (visual orientation discrimination) rather than a simple detection task enabled us to exclude the possible involvement of response/decisional biases. The above evidence suggest that relatively lower-level of neural and perceptual processes are mainly involved in TIVS^15,19^. Findings similar to TIVS have been reported in mice, namely that sounds or tactile stimuli inhibit neural responses to light in the primary visual cortex, and also suppress visually triggered behavioral responses^58^. Intriguingly, the neural responses to visual stimuli in mice are reported to increase when the responses to the auditory stimuli are inhibited. These findings indicate that inhibitory neural circuits fundamentally exist and work in multisensory processing based on the stimulus/neural intensity^59^. We can assume that spatial discrepancies and discrepancies in stimulus intensity in crossmodal stimuli are essential factors for triggering TIVS. It is highly likely that a functional role for crossmodal perceptual suppressions is to suppress weak or unreliable inputs as perceptual noise in order to establish a coherent and robust perception of the surrounding environment^15,19,20^.

Our results also found a positive relationship between the total AQ score and the magnitude of TIVS when the visual and tactile stimuli were co-localized (Fig. 3). It has been demonstrated that the magnitude of visuo-tactile interaction is weaker in individuals with ASD diagnoses and in those with higher autistic traits^43,51–53^. Thus, we predicted that the magnitude of TIVS would weaken as autistic traits became more prominent. The results of the current study, on the other hand, indicate that the magnitude of TIVS become stronger in parallel with stronger autistic traits. The particular characteristics of tactile processing related to ASD may be related to this finding. It has been suggested that proprioceptive sensation is stronger in individuals diagnosed with ASD^60,61^. This phenomenon has also been shown to affect the manner of visuo-tactile interactions. For example, it has been reported that the rubber-hand illusion, in which the simultaneous stimulation of a participant’s hidden hand and a visible rubber hand creates the illusory ownership of the rubber hand^62^, occurs less persistently in children diagnosed with ASD than in children without ASD^51^. Similarly, adults diagnosed with ASD^52,53^ and those with higher autistic traits^43^ are reported to have specific behavioral patterns for and weaker magnitudes of responses in the rubber-hand illusion. These studies suggest that, in individuals with ASD and those with higher autistic traits, a stronger dependency on their proprioceptive sensation modulates visuo-tactile interactions. It is likely that the individuals with higher autistic traits in the current study also tended to rely on their proprioceptive sensation. Consequently, an elevated perception of the tactile stimulus strengthened the TIVS magnitude for these participants.

Given that individuals with higher autistic traits place a greater weight on proprioceptive sensation, this might also explain why the relationship between the TIVS magnitude and the AQ score was detected when the visual and tactile stimuli were co-localized. It has been noted that the spatial range of visuo-tactile interactions is malleable and subject to change. For example, tool use modulates the spatial range of visuo-tactile interactions, at both behavioral and neural levels^48^. As mentioned above, an ideal situation for the occurrence of TIVS seems to be one in which the visual and tactile stimuli are not consistent in space. This in turn suggests that the visuo-tactile stimuli in the co-localized condition may be perceptually processed as spatially inconsistent by individuals with higher autistic traits. Consistent with this idea, it has been suggested that individuals diagnosed with ASD have narrower peripersonal spaces and steeper self-other boundaries^63^. In addition, individuals diagnosed with ASD are reported to have an altered spatial property for visuo-tactile processing^64^. In individuals diagnosed with ASD, numerical judgments of tactile stimuli presented on a participant’s hand are affected by visual stimuli even when the visual stimuli are presented on the contralateral side of the hand. This visual effect on the tactile judgment was limited to the ipsilateral side in individuals without ASD diagnoses. We should note that in the above study, the visual distractor stimulus was presented at a clearly perceptible level, while the tactile target stimuli were presented at the discrimination threshold level, and the observed effects were not discernable at the response, attentional, or perceptual levels of processing. Nevertheless, the findings of the pervious study suggest that autistic traits have modulatory effects on the spatial range of the visuo-tactile interactions. Here we demonstrated that TIVS occurred in a wider spatial range of visuo-tactile interactions in participants with higher autistic traits. Considering that a stronger perceptual weight on proprioception may exist in individuals with higher autistic traits, this effect may be based not on an enlargement, but rather on a reduction of the spatial range for visuo-tactile interactions. Atypical neural processing similar to that observed in individuals diagnosed with ASD^44–46^ may underlie the altered responses associated with autistic traits in individuals without ASD diagnoses in the current study.

Consistent with those of our previous study^15^, the findings of the present study indicate that crossmodal perceptual suppression from the tactile to the visual modality is likely to be predominant at an ipsilateral but spatially distinct position. This aspect should be investigated in greater detail by manipulating the distance between the visual and tactile stimuli at ipsilateral positions in a future study. Our results also suggest that the magnitude of the suppressive visuo-tactile interaction at the co-localized position could vary based on the degree of the autistic traits, due to differences in the size of the spatial range for visuo-tactile interactions and the manner of processing of tactile perception/proprioception. The validities of these proposed ideas should be examined by directly investigating the spatial range of visuo-tactile interactions and the perceptual strength of tactile perception/proprioception in future studies. In addition, the positive relationship between the magnitude of TIVS and the autistic traits implies the existence of stronger suppressive neural interactions from tactile to visual areas in the brain^19^ in individuals with higher autistic traits. Thus, a future study should be performed to investigate the neural processing underlying TIVS, together with the measurement of autistic traits.

## Methods

### Ethics statement

The experimental procedures were approved by the local ethics committee of Rikkyo University, and were performed in accordance with the approved guidelines and the Declaration of Helsinki. Informed consent was obtained from each participant before conducting the experiments.

### Participants and apparatus

Fifty-five university students (18–23 years old; mean: 20.10; standard deviation [SD]: 1.40; 35 females) participated in the experiment. All participants had self-reported normal or corrected-to-normal vision, as well as normal hearing and touch. They were naive to the purpose of the experiment. Fifty participants were strongly right-handed (+50 ≤ laterality quotient [LQ] ≤ +100), while three and two participants were judged to be moderately (LQ = −12.5, −20, and 25) or strongly (LQ = −71 and −79) left-handed, as assessed using the Edinburgh Inventory^65^.

We placed a linearized LCD display (LG, D2342) horizontally on a hand-made aluminum stand in front of the participants (Fig. 1). Visual stimuli were presented on the display, which had a resolution of 1360 × 768 pixels and a refresh rate of 60 Hz. The viewing distance was 29.5 cm. Tactile stimuli were presented through an audio interface (Roland, EDIROL FA-66) and a vibrating device (Eishindenki, Attachable Speaker M-PZT-02) at 91 dBA. The tactile stimuli were amplified by an amplifier (Eishindenki, ED-PZT01B). In order to mask the sound emitted by the vibrating device, white noise bursts (75 dBA) were generated digitally (sampling frequency 44.1 kHz) and delivered through headphones (Pioneer SE-M531). A customized PC (Dell Precision T3500) and MATLAB (MathWorks, Inc.) with the Psychophysics Toolbox^66,67^ were used to control the experiment. A numeric keypad was used to record the responses. We confirmed that the onset of the visual and tactile stimuli was synchronized using a digital oscilloscope (OWON, PDS5022TFT). All experiments were conducted in a dark room.

### Stimuli

We presented the visual and tactile stimuli in a similar manner to our previous study^15^. The tactile stimulus was presented as a vibration (300 Hz sinusoidal burst) for 200 ms with 1 ms of cosine ramp at the onset and offset. The vibrating device was attached to the participant’s index finger and placed on the palm of their left hand. A fixation point consisting of a bull’s eye and crosshair (0.88° × 0.88°, 0.15 cd/m^2^)^68^ was presented at the center of the display. A gray ring (3.53° in diameter, 12.73 cd/m^2^) was presented on a gray background (45.01 cd/m^2^). The ring was presented to the lower left (ipsilateral to the tactile stimulus at the left hand) (ipsi-lower condition), upper left (ipsi-upper condition), or the lower right (contralateral to the tactile stimuli) (cont-lower condition) of the fixation point. Each position was separated from the fixation point by 7.05° of horizontal and vertical distance. A Gabor patch (1.76° × 1.76°, 2.5 cycle/degree, σ = 0.5°, 180° of phase angle) was presented as a target for 50 ms inside the ring. The stripes of the target stimulus were slanted either to the left (−45°) or right (+45°). The target’s contrast (Weber contrast) was either 0.05, 0.10, 0.15, 0.20, 0.25, or 0.30. The ring color was changed from gray to black (0.15 cd/m^2^) before the target presentation.

### Procedure

At the beginning of each session, the gray ring appeared in the ipsi-lower position. The participants placed his or her hand with their palm facing upward on a small plastic box on the top of which a soft urethane sheet was mounted. We asked the participant to place the index finger of their left hand, to which the vibrating device was attached, just below the ring position. The experimenter confirmed that the positions of the ring and hand were consistent. The gray ring then appeared at one of three positions (ipsi-lower, ipsi-upper, and cont-lower spatial conditions) to notify the participants where the visual target was to be presented in the trial. In each trial, after the presentation of the fixation point and the gray ring for 1000 ms, the ring color changed from gray to black for 50 ms as the cue for target onset. The visual target was then presented for 50 ms. Concurrently, the tactile stimulus was presented for 200 ms (touched condition). The onset timing of the visual and tactile stimuli was consistent. A trial without the tactile stimulus was also included to obtain baseline measurements (without-touched condition). After the target presentation, the gray ring reappeared and the participants were asked to judge whether the target was perceived as tilting to the left or right. This experiment consisted of both practice and test sessions. The practice session consisted of 10 trials wherein the visual target was presented with maximum contrast (0.30) at one of the target positions without the presentation of the tactile stimulus: Target’s orientations (2) × Repetitions (5). The main test session consisted of 168 trials for each spatial condition (504 trials in total): Tactile conditions (2) × Target’s contrasts (6) × Target’s orientations (2) × Repetitions (7). Each spatial condition was introduced in a blocked design and the order of the conditions was randomized and counterbalanced among the participants. The order of the other conditions was randomly assigned in each trial and counterbalanced among the participants. After the experiment, we measured the participants’ autistic traits using the Japanese version of the AQ. The results of the AQ have been confirmed to be similar between in Japan and the United Kingdom, suggesting that the reliability and validity of the Japanese questionnaire is sufficient^33^. The AQ is a self-reported questionnaire containing 50 items describing autistic traits. Participants are asked to rate the degree to which the content of each item describes them on a 4-point Likert scale (“definitely agree,” “slightly agree,” “slightly disagree,” and “definitely disagree”). Items that the participants rated as 1 or 2 (or 3 or 4, for reversed items) were assigned a score of 1 point. The scores of all 50 items were summed to calculate the total AQ score.

### Data Analysis

We plotted the proportion of correct responses for each target’s contrast. The proportion of correct responses (*p*) was then fitted by a cumulative density function of a Gaussian distribution in each condition for each participant using the following formula using MATLAB:

$$p(t)=({p}_{max}-{p}_{min}){\int }_{-\infty }^{t}\frac{1}{\sqrt{2\pi \sigma }}exp[\frac{-{(\tau -d)}^{2}}{2{\sigma }^{2}}]d\tau +{p}_{min}$$

Here *t*, *d*, *σ*, *p*_max_, and *p*_min_ denote the target contrast, size of the horizontal transition, resolution, and upper and lower asymptotes of the correct response rates, respectively. We estimated the 75% response point (d) as the discrimination threshold in each condition. We subtracted the estimated threshold of the without-touched condition from that of the touched condition to obtain the index of the magnitude of TIVS for each spatial condition. Larger values indicate stronger TIVS effects. We did not focus on discrimination sensitivity (e.g., sigma of the fitted function) because TIVS has shown to be the change in perceptual threshold^15^.

We excluded 20 participants from the analyses because their data were not well fitted (R^2^ < 0.20) in at least one of the six conditions (3 target positions and 2 tactile conditions). Four participants, whose estimated thresholds were lower or higher than the contrast range defined in the experiment (0.05–0.30) in at least one of the six conditions, were also excluded. We then excluded data from three participants because they were greater than ±2 SD away from the mean in at least one of the six conditions (Supplementary Fig. S1). Consequently, R^2^ values for the analyzed data (N = 28, 18–22 years old; mean: 20.23; SD: 1.30; 17 females) ranged from 0.22 to 1.00, with a mean (SD) of 0.86 (0.15). The mean (SD) total AQ score of the excluded participants was 20.4 (7.19).

The main purpose of the statistical analyses was to investigate the magnitude of the TIVS independently for each spatial condition, rather than compare the magnitudes of the TIVS among the spatial conditions with the null hypnosis assuming that the magnitudes for all spatial conditions were equivalent. Thus, we performed planned one-tailed *t*-tests between the magnitude of the TIVS and zero without a correction of multiple comparisons. Correlation analyses (two-tailed) of the magnitude of TIVS and the total AQ score were also performed independently for each spatial condition. We also a performed the repeated measures ANOVA with the spatial conditions and tactile conditions as factors to assess the general tendencies for the different conditions. We used JASP (version 0.9)^69^ for data analysis.

## Electronic supplementary material

Supplementary figure S1

## Data Availability

The datasets generated and/or analyzed in the current study are available from the corresponding author upon reasonable request.
