# Supplementary material for: Effects of spatial consistency and individual difference on touch-induced visual suppression effect
Source: Sci Rep. 2018 Nov 19;8:17018. doi: 10.1038/s41598-018-35302-w (PMC6242815; doi:10.1038/s41598-018-35302-w)
Supplement: Supplementary file 1 — Supplementary figure S1 [file 41598_2018_35302_MOESM1_ESM.docx]

## Supplementary Information

## Effects of spatial consistency and individual difference on touch-induced visual suppression effect

Souta Hidaka^1, #^, Yosuke Suzuishi^1, #^, Masakazu ide^2^, and Makoto Wada^2, #^

1. Department of Psychology, Rikkyo University, 1-2-26, Kitano, Niiza-shi, Saitama, 352-8558 Japan.

2. Developmental Disorders Section, Department of Rehabilitation for Brain Functions, Research Institute of National Rehabilitation Center for Persons with Disabilities, 4-1, Namiki, Tokorozawa-shi, Saitama, 359-8555 Japan.

# These authors equally contributed to the work.

*Corresponding authors:

Souta Hidaka

E-mail: hidaka@rikkyo.ac.jp

Address: Department of Psychology, Rikkyo University, 1-2-26, Kitano, Niiza-shi, Saitama, 352-8558 Japan.

Makoto Wada

E-mail: wada-makoto@rehab.go.jp

Address: Developmental Disorders Section, Department of Rehabilitation for Brain Functions, Research Institute of National Rehabilitation Center for Persons with Disabilities, 4-1, Namiki, Tokorozawa-shi, Saitama, 359-8555 Japan


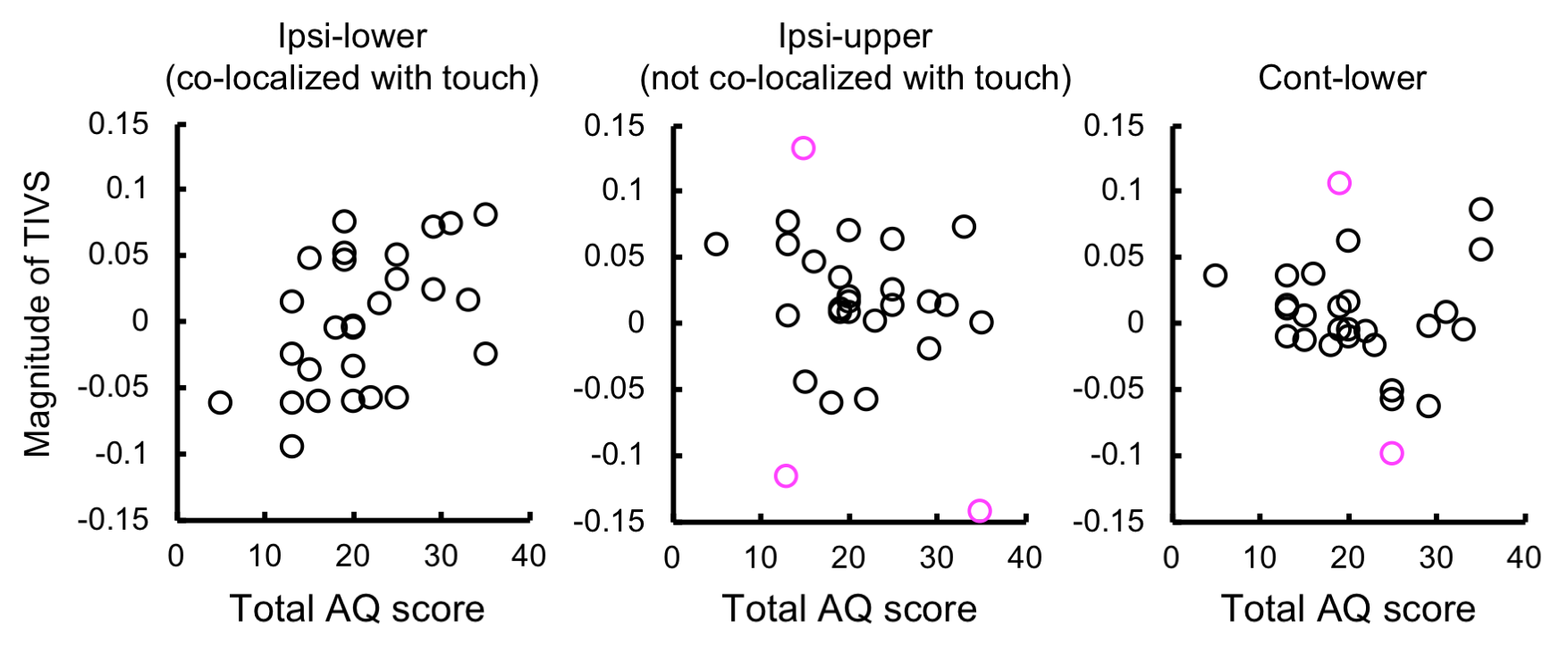


Supplementary figure S1. Scatter plot depicting the magnitudes of TIVS and the total AQ score for each spatial condition. The marks colored magenta indicates the data which exceeded ±2 SD.
